# One of Three Pex11 Family Members Is Required for Peroxisomal Proliferation and Full Virulence of the Rice Blast Fungus *Magnaporthe oryzae*

**DOI:** 10.1371/journal.pone.0134249

**Published:** 2015-07-28

**Authors:** Jiaoyu Wang, Ling Li, Zhen Zhang, Haiping Qiu, Dongmei Li, Yuan Fang, Hua Jiang, Rong Yao Chai, Xueqin Mao, Yanli Wang, Guochang Sun

**Affiliations:** 1 State Key Laboratory Breeding Base for Zhejiang Sustainable Pest and Disease Control, Institute of Plant Protection Microbiology, Zhejiang Academy of Agricultural Sciences, Hangzhou, China; 2 School of Agricultural and Food Sciences, Zhejiang Agriculture and Forest University, Hangzhou, China; 3 College of Chemistry and Life Sciences, Zhejiang Normal University, Jinhua, China; Fujian Agriculture and Forestry University, CHINA

## Abstract

Peroxisomes play important roles in metabolisms of eukaryotes and infection of plant fungal pathogens. These organelles proliferate by de novo formation or division in response to environmental stimulation. Although the assembly of peroxisomes was documented in fungal pathogens, their division and its relationship to pathogenicity remain obscure. In present work, we analyzed the roles of three Pex11 family members in peroxisomal division and pathogenicity of the rice blast fungus *Magnaporthe oryzae*. Deletion of *MoPEX11A* led to fewer but enlarged peroxisomes, and impaired the separation of Woronin bodies from peroxisomes, while deletion of *MoPEX11B* or *MoPEX11C* put no evident impacts to peroxisomal profiles. *MoPEX11A* mutant exhibited typical peroxisome related defects, delayed conidial germination and appressoria formation, and decreased appressorial turgor and host penetration. As a result, the virulence of *MoPEX11A* mutant was greatly reduced. Deletion of *MoPEX11B* and *MoPEX11C* did not alter the virulence of the fungus. Further, double or triple deletions of the three genes were unable to enhance the virulence decrease in *MoPEX11A* mutant. Our data indicated that *MoPEX11A* is the main factor modulating peroxisomal division and is required for full virulence of the fungus.

## Introduction

Peroxisomes, single membrane-bounded organelles, present in all eukaryotes except the amitochondrial parasites *Entamoeba* and *Giardia* [1,2]. The functions of the organelles commonly include fatty acid β-oxidation and hydrogen peroxide metabolism [3,4], and in recent years, expand to various specific roles [5]. Peroxisomes are inducible by culturing and cellular environments, and this inducibility was proved to be essential for metabolism and survival of the organisms [6,7]. In response to environmental stimulation, peroxisomes proliferate rapidly by the *de novo* formation from the endoplasmic reticulum (ER), or alternatively, by the division of pre-existing peroxisomes [8–10]. Both of the two pathways require a series of specific proteins, designated commonly as peroxins [11].

As the beginning of the peroxisomal division, the peroxisomal membranes are defined spatiotemporally and grow polarly, leading to extensively change in their shapes to form protrusion and elongation. The matrix proteins are subsequently imported into the elongated area, coinciding with recruitment of the fission machinery and constriction of the organellar membrane. Finally, the organelles are separated into individual daughter peroxisomes under the regulation of fission factors (e.g. Dnm1, Vps1, Drp1) shared with mitochondrial fission machinery [12–17]. Pex11 family proteins, a category of peripheral peroxisomal membrane proteins (PMPs), play key roles in peroxisomal division [12,17,18]. The protein levels of Pex11 modulate the numbers and sizes of peroxisomes: absence of Pex11 leads to reduction of peroxisomal numbers while overproduction promotes peroxisomal proliferation [16,19,20]. Pex11 was demonstrated to act as membrane elongation factor, which directed the peroxisomal deformation and elongation prior to the fission steps [13,21–24]. Pex11 orthologs are present in all eukaryotes containing peroxisomes. *Saccharomyces cerevisiae* has a single Pex11, whereas human cells contain three Pex11 isoforms, of which, Pex11α is responsible for peroxisome proliferation in response to external stimuli, while Pex11β is required for constitutive peroxisome proliferation [25–27]. In addition to Pex11, Pex25 and Pex27 contribute to peroxisomal proliferation in *S*. *cerevisiae* [28–30].

Peroxisomes play vital roles as well in filamentous fungi, involving metabolisms of various carbon and nitrogen sources [31–37]. Basing on sequence similarity, most of filamentous fungi are thought to contain three or more Pex11 isoforms [38]. In *Penicillium chrysogenum*, Pex11 was demonstrated as a major controller for peroxisomal number and size, Pex11C played minor roles as well, whereas Pex11B was likely dispensable in peroxisomal proliferation [39]. Five Pex11 isoforms were detected in *Aspergillus oryzae*, but only Aopex11-1 contributes to peroxisomal function and proliferation [40]. Additionally, Aopex11-1 is also required for the separation of Woronin body, a unique organelle in Pezizomycotina, from the peroxisomes [40]. Thus the functions of Pex11 isoforms in fungi are complicated and species-specific. However, Pex11 isoforms in filamentous fungi were only investigated in *A*. *oryzae* and *P*. *chrysogenum* so far and left much to be understood.

On the other hand, peroxisomes were found to be crucial for host invasion of plant pathogenic fungi [32–34,41]. Deletion of *PEX6* damaged peroxisomal metabolism and impaired the fungal infection greatly in *Colletotrichum lagenarium* and *Magnaporthe oryzae* [32,42,43]. *PEX13* was also found indispensable for the infection of *Colletotrichum orbiculare* [44]. *PEX5* and *PEX7* are both required for development and pathogenicity in *M*. *oryzae*, while *PEX5* is likely more contributional [34,41]. *PEX5* and *PEX6*, other than *PEX7* are crucial to the survival and the virulence in *Fusarium graminearum* [45]. Deletion of *PEX19* led to absence of peroxisomal structures in *M*. *oryzae* and resulted in severer damages to nutrition utilization and pathogenicity than the deletion of *PEX5* or *PEX7* [37]. Previously, we found the numbers of peroxisomes increased sharply at the early stage of conidial germination in *M*. *oryzae* [46]. This suggests the involvement of peroxisomal proliferation in the infection of the fungus. All the *PEX* genes investigated in pathogenic fungi so far are involved in the assembly of peroxisome, however, no direct data indicated the roles of peroxisomal proliferation in fungal pathogenicity.

To better understand the peroxisomal proliferation in filamentous fungi and its contribution to fungal pathogenicity, in present work, we characterized the three predicted *PEX11* genes in *M*. *oryzae*, *MoPEX11A*, *MoPEX11B* and *MoPEX11C*. Our data showed that deletion of *MoPEX11A* altered the number and size of peroxisomes. Δ*mopex11A*, other than Δ*mopex11B* and Δ*mopex11C*, exhibited obvious defects in fungal development and reduced the virulence greatly. These findings indicate that *MoPEX11A* is a main factor in modulating the peroxisomal proliferation in *M*. *oryzae*, and peroxisomal proliferation is indispensable for the full virulence of the fungus.

## Material and Methods

### Strains, cultivation and transformation

*M*. *oryzae* wild type Guy11 [47] and all transformants were routinely cultured on complete medium (CM) at 28°C for 3 to 14 days [48,49]. To isolate genomic DNA, the fungus was cultivated in liquid CM for 3 days. Lipid medium, glucose medium and sodium acetate medium were prepared as described [34]. All fungal transformants were generated by *Agrobacterium tumefaciens*-mediated transformation (*At*MT) as described [50]. CM plates containing 250 μg/ml hygromycin B (Roche, Mannheim, Germany), 200 μg/ml glufosinate–ammonium (Sigma, St Louis, MO, USA) or 800 μg/ml G418 (Sigma) and defined complex medium (DCM; 0.16% yeast nitrogen base without amino acids, 0.2% asparagine, 0.1% ammonium nitrate and 1% glucose, pH 6.0 with Na_2_HPO_4_) [32] containing 100 μg/ml chlorimuron ethyl (Sigma) were used for screening the corresponding transformants. Cell wall integrity was tested by growing the strains on CM supplemented with 100 μg/ml Congo red. The tolerance of the strains to ROSs was evaluated by the growth on CM containing 2.5 or 5.0 mM H_2_O_2_ or 1 mM methyl viologen.

### Sequence analysis

The Pex11 homologues were identified by searching from the Genbank or the fungal genome database (http://www.broadinstitute.org/scientific-community/data). The real coding sequences of *MoPEX11A*, *MoPEX11B* and *MoPEX11C* were determined by PCR amplification using the cDNA revers-transcripted from the total RNA of the fungus as template. Sequence alignments were performed using the Clustal W method, and imported into the software GeneDoc 2.0 (http://genedoc.software.informer.com/download/) for type setting and into MEGA version 5.0 (http://www.megasoftware.net/) to establish the phylogenetic trees.

### Nucleic acid manipulation

The genomic DNA was isolated using the cetyl trimethyl ammonium bromide method [48]. Total RNA was prepared using the Trizol reagent (Invitrogen, Carlsbad, CA, USA), and used to synthesize the cDNA using AMV Reverse Transcriptase (Takara Bio, Otsu, Japan). PCR, Restriction digestion, gel electrophoresis and ligation reactions were performed using standard procedures. Transcript abundance was analyzed by quantitative PCR on the 7500 Fast Real-Time System (Applied Biosystems, Foster, CA, USA) with the β-tubulin gene (MGG00604) for normalization. Southern blotting was performed using the digoxin high-prime DNA labeling and detection starter kit I (Roche).

### Construction of fluorescent fusions

The GFP fragment was amplified with the primer set GFP-*Eco*RI /GFP-*Xba*I and pBMGFP [46] as template, and inserted into *Eco*RI/*Xba*I sites of p1300Bar [46] to generate p1300BarGFP. To monitor the expression of *MoPEX11A*, *MoPEX11B* and *MoPEX11C*, at least 1.5 kb promoter regions were amplified using the primer sets 11Aproup/11Aprodn, 11Bproup/11Bprodn and 11Cproup/11Cprodn. The promoters of *MoPEX11A* and *MoPEX11C* were introduced into p1300BarGFP by *Pst*I/*Bam*HI digestion, and the promoter of *MoPEX11B* was introduced into p1300BarGFP by *Pvu*I/*Xba*I digestion, to generate the GFP expression vectors p1300B11APGFP, p1300B11BPGFP and p1300B11CPGFP, respectively.

To visualize the peroxisomes, the vectors containing fluorescent proteins fused to peroxisomal targeting signal 1 (PTS1) or PTS2, p1300BMGFPA (GFP-PTS1), p1300BMGFPB (GFP-PTS2) and p1300NMRFPA (DsRed-PTS1) [46] were used as peroxisomal markers. The Woronin body major protein Hex1 was fused to mCherry using similar procedures to generate pNMCH-HEX1, as the fluorescent markers for Woronin body.

### Gene deletion and mutant complementation

For gene deletion, at least 1.5 kb upstream and downstream fragments for each gene were amplified from genomic DNA and inserted into p1300-KO [37] to generate the gene replacement vectors pKO11A-HPH, pKO11B-HPH and pKO11C-HPH, respectively, which were then integrated into the *M*. *oryzae* Guy11 strain. The transformants resistant to hygromycin B were harvested and screened preliminarily by genomic PCR with primer sets 11Acds2/11A cds3, 11Bcds2/11Bcds3 and 11Ccds2/11Ccds3, respectively. The potential gene deletion mutants of them were further confirmed by Southern blotting. For double and triple deletions, pKO11A-HPH and pKO11B-HPH were modified by substituting the hygromycin resistant gene (*HPH*) with the chlorimuron-ethyl resistant gene (*SUR*) and the G418 resistant gene (*NEO*) to generate pKO11A-SUR and pKO11B-NEO respectively. The gene deletion vectors carrying different resistance were integrated into confirmed single or double gene deletion mutants by different combinations to perform the double and triple gene deletion. The generation procedures of the mutants were shown in S1 Fig

For mutant complement, the genomic fragments containing full lengths of ORFs, 1.5 kb upstream and 0.5 kb downstream of the *MoPEX11A* and *MoPEX11B* were amplified and inserted into p1300BAR [46] to generate complementary vectors p1300BAR-11Acom and p1300BAR-11Bcom, respectively, which were integrated into single or triple deletion mutants (S1 Fig). The resulting transformants were picked up by glufosinate–ammonium resistance and checked by genomic PCR. The candidate complementary strains from which were further confirmed by detecting the transcripts of corresponding genes using quantitative PCR with β-tubulin gene (MGG00604) as housekeeping gene control. For each gene, two confirmed revertants were used in phenotypical analysis.

All the primers used in this study were listed in Table 1.

**Table 1:** Primers used in this study.

Name	Sequence (5’-3’)	To amplify
11-1CDS2	TCCCCCGGGTTGGTCGCCGACGCCCTCGTATAC	CDS of *MoPEX11A*
11-1CDS3	TCCGAGCTCTGAAAGACCGGTTTGAGCAGCCAC	CDS of *MoPEX11A*
11-2CDS2	TCCCCCGGGTTGCAGCAATTCATTAGATTCAGT	CDS of *MoPEX11B*
11-2CDS3	TCCGAGCTCGCTCGTATATACCTCAACTCACCC	CDS of *MoPEX11B*
11-3CDS2	TCCCCCGGGTTGACGTCTTCAGCCGAAATCACA	CDS of *MoPEX11C*
11-3CDS3	CTGCAGAACCACCATGTTGGTAGAAAACATGCAGAAAATTGAAC	CDS of *MoPEX11C*
11-1KO1	GAAGAGACGGTGGGGACGAGGTTG	5’ flank of *MoPEX11A*
11-1KO2	CGGGATCCTTTGGGCTATTTGGTTTGTTGTTT	5’ flank of *MoPEX11A*
11-1KO3	CTCTGACCTGTGCGATCTGACCAT	3’ flank of *MoPEX11A*
11-1KO4	CGGAATTCCCGACTCCCAAATATCCTGTGACC	3’ flank of *MoPEX11A*
11-2KO1	CGGAATTCCGGCTTTGCTGTCGGTTGGGAGTAT	5’ flank of *MoPEX11B*
11-2KO2	GGGGTACCAAAATGAATGTGGATGTTGAGAGC	5’ flank of *MoPEX11B*
11-2KO3	TTCAAGCGGCTGTATTTTAGACGA	3’ flank of *MoPEX11B*
11-2KO4	TGGGGCAGCAGGAGCAGGCAACT	3’ flank of *MoPEX11B*
11-3KO1	CCCAAGCTTATTAGGTGGTCGGCGCGGAGTTGT	5’ flank of *MoPEX11C*
11-3KO2	CGGGATCC TGGTGGGTTTGTATGGTGGACGAA	5’ flank of *MoPEX11C*
11-3KO3	CGTCGCCTGGTCGCCTGTCATCAT	3’ flank of *MoPEX11C*
11-3KO4	CCAGGGAATAAACCACCACTACCA	3’ flank of *MoPEX11C*
P11A- f	AACTGCAGGAAGAGACGGTGGGGACGAGGTTG	promoter of *MoPEX11A*
P11A- r	CGGGATCCTTTGGGCTATTTGGTTTGTTGTTT	promoter of *MoPEX11A*
P11B- f	ATCGATCGGGCTTTGCTGTCGGTTGGGAGTAT	promoter of *MoPEX11B*
P11B- r	GCTCTAGATTTGCAGAATGAGATGAGAAAATG	promoter of *MoPEX11B*
P11C- f	AACTGCAGATTAGGTGGTCGGCGCGGAGTTGT	promoter of *MoPEX11C*
P11C- r	GCTCTAGATATGGTTGGTGGGTTTGTATGGTG	promoter of *MoPEX11C*
11A-com-f	CGGAATTCGAGAAAAGGGAAGGGGAGGAAAGGA	full length of *MoPEX11A*
11A-com- r	GCTCTAGATCGTAGAAGCGCGGATCTGGTATGT	full length of *MoPEX11A*
q11A-f1	AGGGCAAGAGGATCGCAATT	transcript of *MoPEX11A*
q11A-r1	GCGGTCTTCTTCCATTGTG	transcript of *MoPEX11A*
qPEX3-f3	GTTCTCGCTCTGTTGACCAT	transcript of *MoPEX3*
qPEX3-r3	GTCGCTGCCATAGACAACAT	transcript of *MoPEX3*
qPEX5-f1	ACAACCTTGCCGCCCTAA	transcript of *MoPEX5*
qPEX5-r1	GTGACACGCTTTCGGAGTT	transcript of *MoPEX5*
qPEX7-f2	ACGTTTGACACCAACGAT	transcript of *MoPEX7*
qPEX7-r2	AGGCTTCCCTTTTGTGCT	transcript of *MoPEX7*
qPTH2-f1	AGCTGGTACCTGTCCACTTCCC	transcript of *PTH2*
qPTH2-r1	TTGAAGTTGATGCTGTTCTCGTTG	transcript of *PTH2*
qDNM1-f1	TTGCACAGCCATTTGGTCAC	transcript of *DNM1*
qDNM1-r1	TGCAATGGTTTACCAGCAGA	transcript of *DNM1*
qFIS1-f3	GATGCGGAAACGCCTCTTCAGC	transcript of *FIS1*
qFIS1-r3	CGGATCGCGTACTGGATTTGA	transcript of *FIS1*

The restriction sites used were underlined.

### Pathogenicity tests and infectious structures observation

Two-week-old rice CO39 and 7-day-old barley ZJ-8 were used in pathogenicity tests. The conidia were harvested from 10-day-old *M*. *oryzae* cultures grown on CM and resuspended at 2×10^4^ conidia/ml [48]. For spray inoculation, 2 ml aliquots of the conidial suspension supplemented with 0.25% (w/v) gelatin were sprayed evenly on 15 seedlings. For inoculation on detached leaves, 20 μl aliquots of the conidial suspension or 5 mm mycelial plugs were placed on leaf segments, and incubated at 28°C darkness for 24 h and subsequent 24 h light for 3 days. For inoculation on wounded leaves, the detached barley leaves were firstly scraped with sandpaper to remove the cuticles. To observe infectious structures, the inoculated barley leaves were discolored with lactic acid, heated at 65°C for 2 h, and examined microscopically.

### Measurement of conidial germination, appressorial formation and turgor genesis

Conidia harvested from 10-day-old *M*. *oryzae* cultures grown on CM were resuspended at 1×10^5^/ml. The 50 μl aliquots of conidial suspensions were incubated on a plastic coverslip at 28°C for 48 h to allow the germination and appressoria formation. The appressoria formed at 24 h and 48 h post incubation were used in the incipient-cytorrhysis assay to measure the appressorial turgor as described (34). The experiments were replicated three times with more than 200 appressoria were counted for each strain.

### Fluorescent microscopy and transmission electron microscopy (TEM)

The fluorescence of GFP and RFP (DsRed and mCherry) were detected under a Leica SP2 Confocal System (Mannheim, Germany), with excitation 488 nm, emission 520 nm for GFP and excitation 558 nm, emission 583 nm for RFP. For TEM analysis, the conidia and mycelia collected from *M*. *oryzae* cultures grown on CM were treated and examined under a JEM-1230 electron microscope (JEOL, Tokyo, Japan) as described [51].

## Results

### Pex11 isoforms in M. oryzae

**Fig 1:**
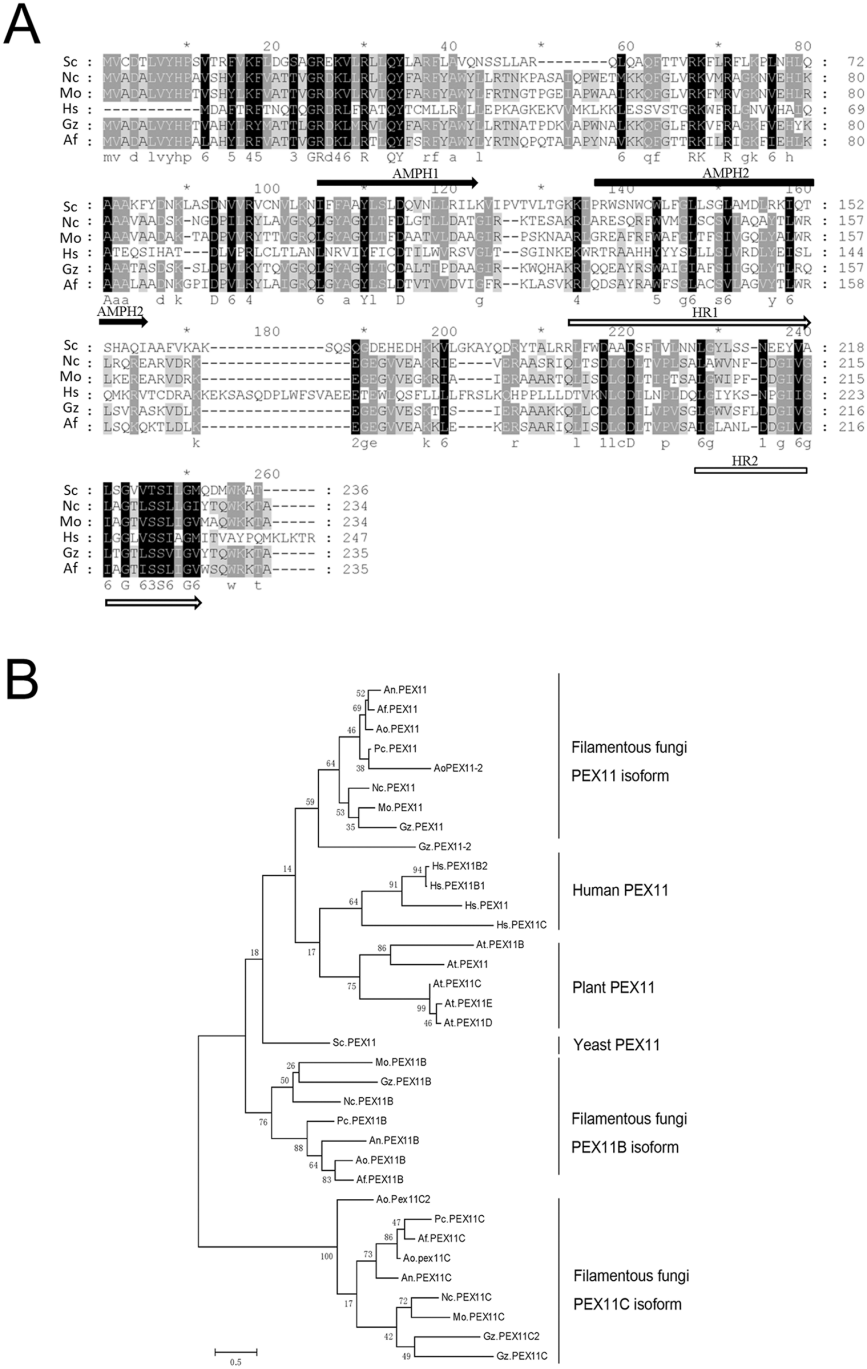
Sequence similarities of Pex11 family members. (A) Sequence alignment of Pex11A proteins from various organisms. The alignment was performed with Clustal W module in MEGA software vision 5 and formatted by GeneDoc (http://www.psc.edu/biomed/genedoc). The identical amino acids are highlighted with black background, the conserved residues with dark gray background, and the similar amino acids with light gray background. The putative amphipathic helices are underlined with black arrows, and hydrophobic regions with hollow arrows. (B) The Neighbor-joining phylogenetic tree of Pex11 proteins constructed using MEGA software. The distance scale represents the differences between the sequences, with 0.1 indicating a 10% difference. Af, *Aspergillus fumigatus*; An, *A*. *nidulans*; Ao, *A*. *oryzae*; At, *Arabidopsis thaliana*; Gz, *Gibberella zeae*; Hs, *Homo sapiens*; Mo, *M*. *oryzae*; Nc, *N*. *crassa*; Pc, *P*. *chrysogenum*; Sc, *S*. *cerevisiae*.

Three hypothetic genes, MGG08896, MGG00648 and MGG05271, were retrieved from *M*. *oryzae* genome as the potential *PEX11* isoforms, and assigned as *MoPEX11A*, *MoPEX11B* and *MoPEX11C*, respectively. cDNA sequencing indicated that *MoPEX11A*, *MoPEX11B* and *MoPEX11C* encode polypeptides of 234-amino-acid, 257-amino-acid and 319-amino-acid respectively, corresponding well with the annotation in genome database. Mopex11A is 31% identical to *S*. *cerevisiae* Pex11 protein and contains two N-terminal amphipathic helices (AMPH) and three C-terminal hydrophobic regions (HR), which are the typical Pex11 structures (Fig 1A). Mopex11B (18.1% identity to Mopex11A) and Mopex11C (20.5% identity to Mopex11A) are relatively remote to Scpex11 in protein levels (S2 Fig). Phylogenetic analysis of Pex11ps from diverse of organisms showed that the fungal Pex11ps are classified into three subclades, of which, the subclade Pex11 (Pex11A) is closely related to mammals and plant Pex11 subclades, while Pex11B and Pex11C subclades are distant (Fig 1B).

#### *MoPEX11A* is up-regulated by lipids and in appressorial differentiation

**Fig 2:**
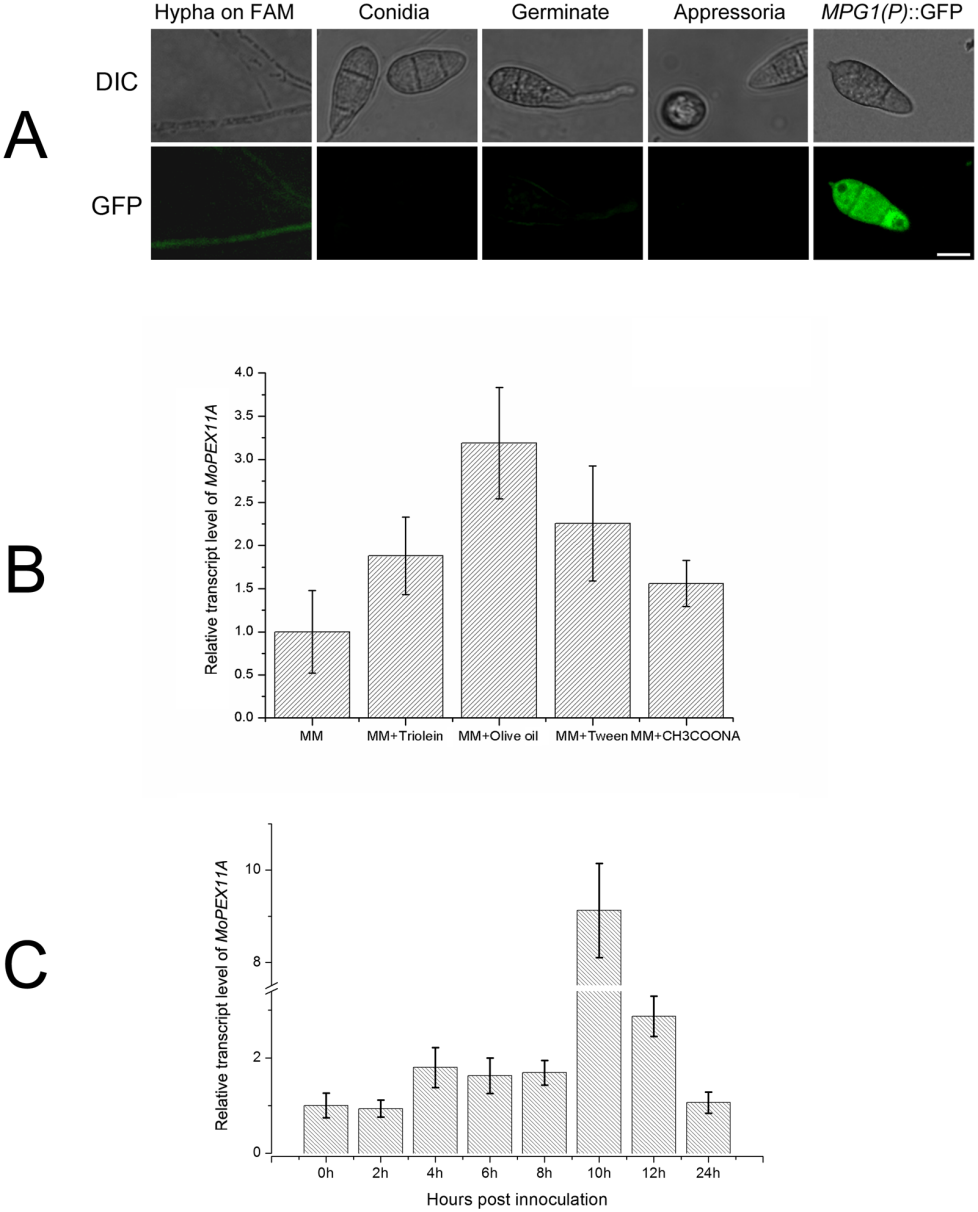
Expression of *MoPEX11A* was assessed by GFP expression strategy and quantitative PCR. (A) The GFP cassette was expressed under the *MoPEX11A* promoter in *M*. *oryzae* wild type and detected by CLSM in various development stages, with the GFP expressed under *MPG1* promoter as a control. FAM (fatty acid media), minimal medium with 1% olive oil as sole carbon source. Bar = 5 μm. (B) Quantitative PCR analysis of the relative transcription levels of *MoPEX11A* in the cultures on the minimal media supplemented with 1% Glucose (MM), Triolein, olive oil, tween 80 or sodium acetate as sole carbon source. (C) The relative transcription levels of *MoPEX11A* during the conidial germination and appressorial formation.

The expression of *MoPEX11A*, *MoPEX11B* and *MoPEX11C* was assessed by detecting the GFP expression under the promoters of the genes. No GFP fluorescence was detectable under any of the promoters in hyphae or conidia of the transformants cultured on CM, neither in the germ tubes or appressoria induced on hydrophobic surface, suggesting that the genes were expressed in very low levels in the basal life activities of the fungus. However, when cultured on olive oil media, the GFP under *MoPEX11A* promoter was able to emit weak fluorescence (Fig 2A). To confirm its expression, the relative transcription of *MoPEX11A* in the wild type strain was assessed by quantitative PCR, and indicated that the *MoPEX11A* could be up-regulated by Triolein, olive oil, Tween 80 or sodium acetate (Fig 2B). Additionally, the transcription of *MoPEX11A* was found increasing during conidial germination and appressorial induction, with a peak at 10 hours post inoculation (hpi), the key phase of appressorial differentiation (Fig 2C). The expression pattern hints the possibility that *MoPEX11A* play roles in lipid metabolism and pathogenicity.

### *MoPEX11A* is required to the full virulence of *M*. *oryzae*

To reveal the functions of *MoPEX11A*, *MoPEX11B* and *MoPEX11C*, gene deletions were performed. For each gene, more than 50 hygromycin-resistant transformants were harvest and primarily checked by genomic PCR. From them, six to seven potential gene deletion mutants with one or two random insertion transformants for each gene were further tested by Southern blotting (S3 Fig). The confirmed gene deletion mutants A21-12, B5-14 and C1-1 were selected to represent the knock mutants of *MoPEX11A*, *MoPEX11B* and *MoPEX11C* in phenotypical analysis, and named KO11A, KO11B and KO11C respectively. To ensure the phenotypes of the mutants attribute really to gene deletion, the genomic fragment covering the full-length of each gene was reintroduced into the responding mutant. The resulting transformants were selected primarily by genomic PCR, and potential complemented transformants of them were confirmed by detection of the gene transcripts. KO11A, KO11B and KO11C, and one of the confirmed complemented transformant for each, were used for phenotypic analysis.

**Fig 3:**
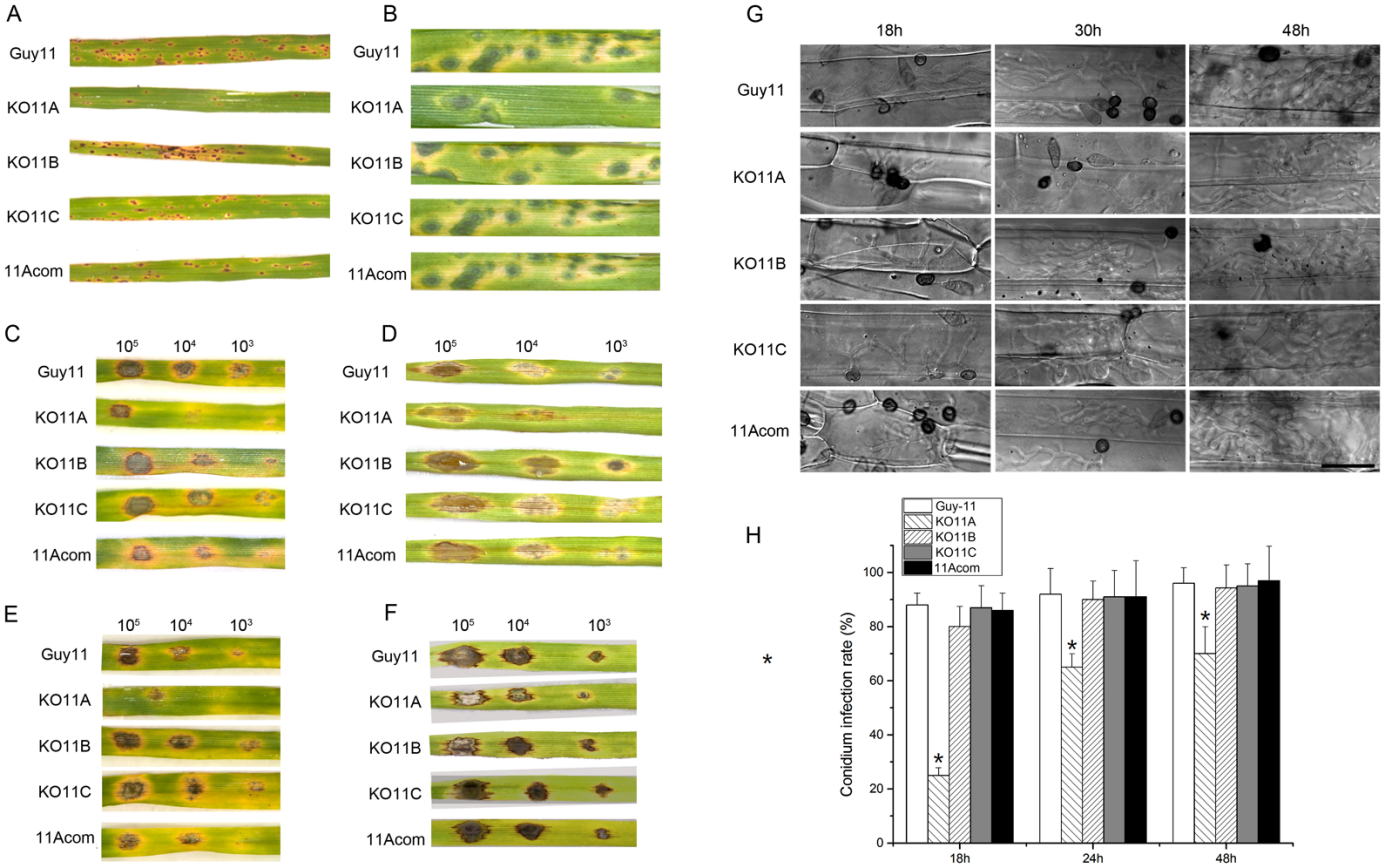
Pathogenicity test of the *MoPEX11* deletion mutants. (A) Spray-inoculation with conidial suspension (1×10^5^ conidia/ml) of the wild type, KO11A, KO11B, KO11C and complemented strains 11A-com on 2-week-old rice cultivar CO39. The symptoms were recorded at 7 days post-inoculation (dpi). (B), Spray-inoculation with conidial suspension (2×10^4^ conidia/ml) on 7-day-old barley cultivar ZJ-8, recorded at 4 dpi. The 20 μl droplets of conidial suspensions in gradient concentrations were inoculated on intact (C) and wounded barley leaves (D) and cultured for 4 days. Droplet-inoculation with conidial suspensions supplied with 2.5% Glucose was performed on intact (E) and wounded barley leaves (F) and cultured for 4 days. (G) Microscopic analysis of the infection process of the mutants. Droplet-inoculated barley leaves were sampled at 18, 30 and 48 h post-inoculation, discolored and examined microscopely. Bar = 40 μm. (H) Statistic analysis of infection rates of the appressoria of the wild type and the mutants. Standard deviations are indicated by the error bars. Asterisks indicate significant differences at p = 0.05.

Unlike the previously documented *pex* mutants in *M*. *oryzae*, KO11A, KO11B and KO11C exhibit no apparent defects in vegetative growth and conidiation on CM (S4 Fig). Nevertheless, the capacity to cause disease of KO11A was greatly reduced. Upon inoculation via various methods, KO11A caused weaker symptoms on rice and barley than the wild type. Meanwhile, the complemented strain of *MoPEX11A* (11Acom) recovered its virulence to equivalent level to that of the wild type (Fig 3A–3F). However, the pathogenicity of KO11B and KO11C were unaffected. Under microscope, most of the KO11A appressoria on the inoculated leaves were found blocked at penetration or differentiation of secondary infectious hyphae (Fig 3G and 3H). More than 83% appressoria of the wild type, KO11B and KO11C penetrated and produced primary infectious hyphae at 18 h post inoculation (hpi), while only 20% appressoria of KO11A did. After 24 hpi, the penetration rate of KO11A rose to 60%, but was still lower than those of the wild type (84%), KO11B (83%) and KO11C (85%). The infectious hyphae of KO11A within the host cells were also retarded compared to the wild type, KO11B and KO11C.

To investigate which developmental defects account for the virulence decrease in KO11A, we compared the conidial germination, appressorial formation and appressorial turgor generation of KO11A, KO11B, KO11C, the wild type and the *MoPEX11A* complemented strain 11Acom (S5 Fig). The germination rate was reduced significantly in KO11A compared with the wild type and 11Acom, while unaltered in KO11B and KO11C (S5A Fig). Although all the mutants form normal-appearanced appressoria (S5B Fig), the formation rate of KO11A was significantly lower than those of KO11B, KO11C, the wild type and 11Acom (S5C Fig). The incipient-cytorrhysis method was used to compare the appressorial turgor. Treated with 0.5, 1 or 2 M glycerol, the appressoria of KO11A collapsed at significantly higher percentages than those of the wild type and 11Acom. However, the collapse rates of the appressoria of KO11B and KO11C were undifferentiated to those of the wild type (S5D Fig). Taken together, *MoPEX11A* is required in appressorial formation, penetration and infectious growth and thus is indispensable for the full virulence of the fungus.

### Deletion of *MoPEX11A* lead to enlarged peroxisomes

**Fig 4:**
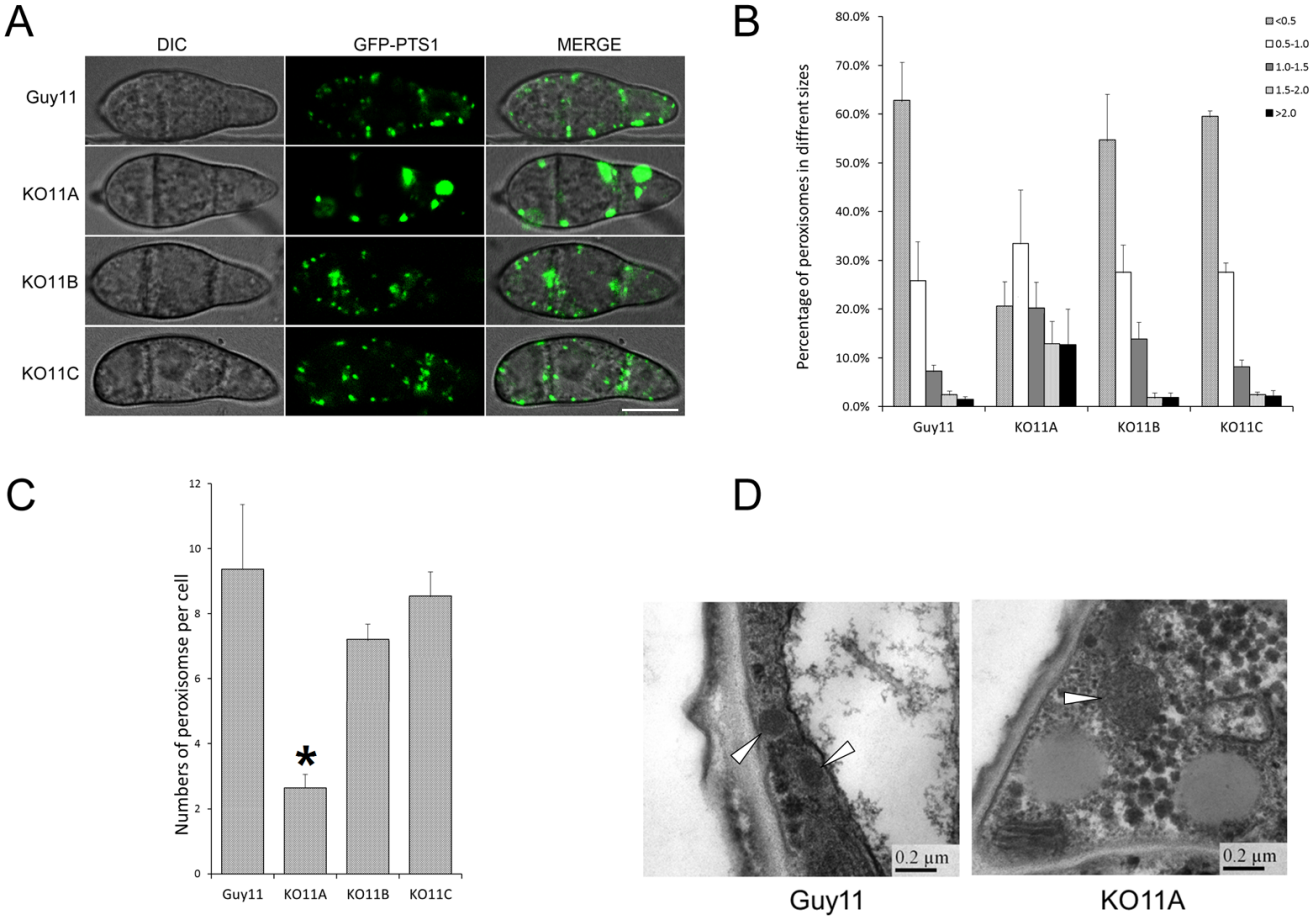
Peroxisomal profiles in the wild type and the *MoPEX11* mutants. (A) CLSM analysis of peroxisomes visualized with GFP-PTS1 in the wild type Guy11, KO11A, KO11B and KO11C. Bar = 5 μm. The sizes (B) and numbers (C) of the peroxisomes in the cells of the strains were statistically compared. Standard deviations are indicated by the error bars. For each strain, more than 100 cells were counted. Asterisks indicate significant differences at p = 0.05. (D) TEM analysis of the Peroxisomes (indicated by arrow heads) in Guy11 and KO11A.

Since peroxisomal metabolism was essential for fungal pathogenicity [42], the virulence decrease of KO11A may suggest that *MoPEX11A* is the main factor in regulation of peroxisomal proliferation. To verify this speculation, we investigated the peroxisomal profiles in the mutants by fluorescent visualization. Under the confocal laser scanning microscopy (CLSM), the fluorescence of GFP-PTS1 (GFP fused with a peroxisomal targeting signal 1) expressed in KO11A, KO11B and KO11C were all presented in dotted pattern, indicating the intact import rotes of peroxisomal matrix proteins (Fig 4A). However, the number of the peroxisomes was greatly reduced in KO11A, and the sizes increased, compared with those in the wild type (Fig 4B and 4C). Additionally, the elongated peroxisomes were increasingly present in KO11A. In contrast, the peroxisomal profile was not significantly affected in KO11B and KO11C. The experiments performed with DsRed-PTS1 (DsRed fused with a peroxisomal targeting signal 1) and GFP-PTS2 (GFP fused with a peroxisomal targeting signal 2) produced the same results (S6 Fig), confirming the alteration of peroxisomal structures. For a further insight, we examined the ultrastructure of the strains. The peroxisomes detected by TEM in KO11A were apparently bigger than those of the wild type (Fig 4D). The alteration of peroxisomal sizes and numbers suggested that *MoPEX11A* is the main regulator in peroxisomal proliferation.

### *MoPEX11A* play roles in separation of Woronin bodies from peroxisomes

**Fig 5:**
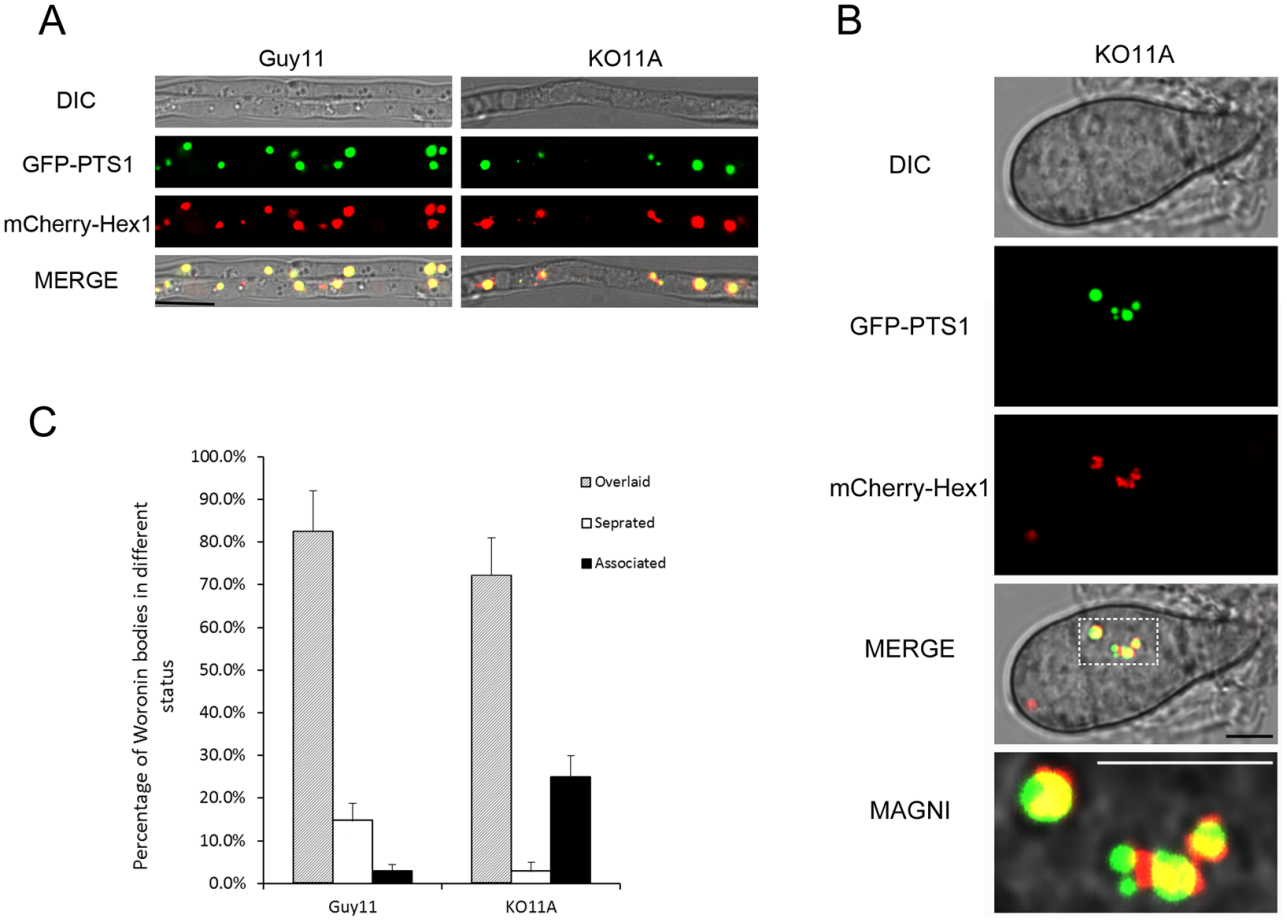
Analysis of Woronin bodies and peroxisomes in Guy11 and KO11A by dual fluorescence strategy. Peroxisomes and Woronin bodies were visualized respectively with GFP-PTS1 and mCherry-Hex1 and detected by CLSM. (A) The profiles of peroxisomes and Woronin bodies in hypha cultured on complete media (CM). (B) Magnified image showing the association of the Woronin bodies to the peroxisomes in KO11A. Bars = 2 μm. (C) Statistically analysis of the Woronin bodies separated, overlaid and associated to the peroxisomes.

Woronin bodies are ascomycete-specific organelles which generate by bud from peroxisomes [52]. To investigate whether the *MoPEX11* genes play roles in Woronin body formation in *M*. *oryzae*, the peroxisomes and the Woronin bodies were visualize simultaneously by GFP-PTS1 and DsRed-MoHex1 (DsRed fused with MoHex1, the woronin major protein in *M*. *oryzae*). In both of the wild type and KO11A, the GFP-PTS1 and DsRed-MoHex1 were distributed in punctate patterns and partially overlaid, indicating that the peroxisomal targeting of Hex1 is unblocked by the deletion of *MoPEX11A* (Fig 5A). Meanwhile, the independent red and green dots could be found in the wild type and KO11A cells, which represent the separated Woronin bodies and the peroxisomes defecating off the Hex1 proteins. Besides, some red puncta were found non-overlaying but associating closely with the green ones, which demonstrated the Woronin bodies undergoing the separation process (Fig 5A and 5B). However, the separated Woronin bodies in KO11A were present in significantly reduced percentage than in the wild type; but instead, more Woronin bodies were arrested at separation steps and remained associating together with peroxisomes (Fig 5C). Collectively, our data suggested that *MoPEX11A* is required in the division of peroxisomes, and as well, in the separation of Woronin bodies from peroxisomes.

### Deletion of *MoPEX11A* reduced the lipids degradation and weaken the cell wall of the fungus

**Fig 6:**
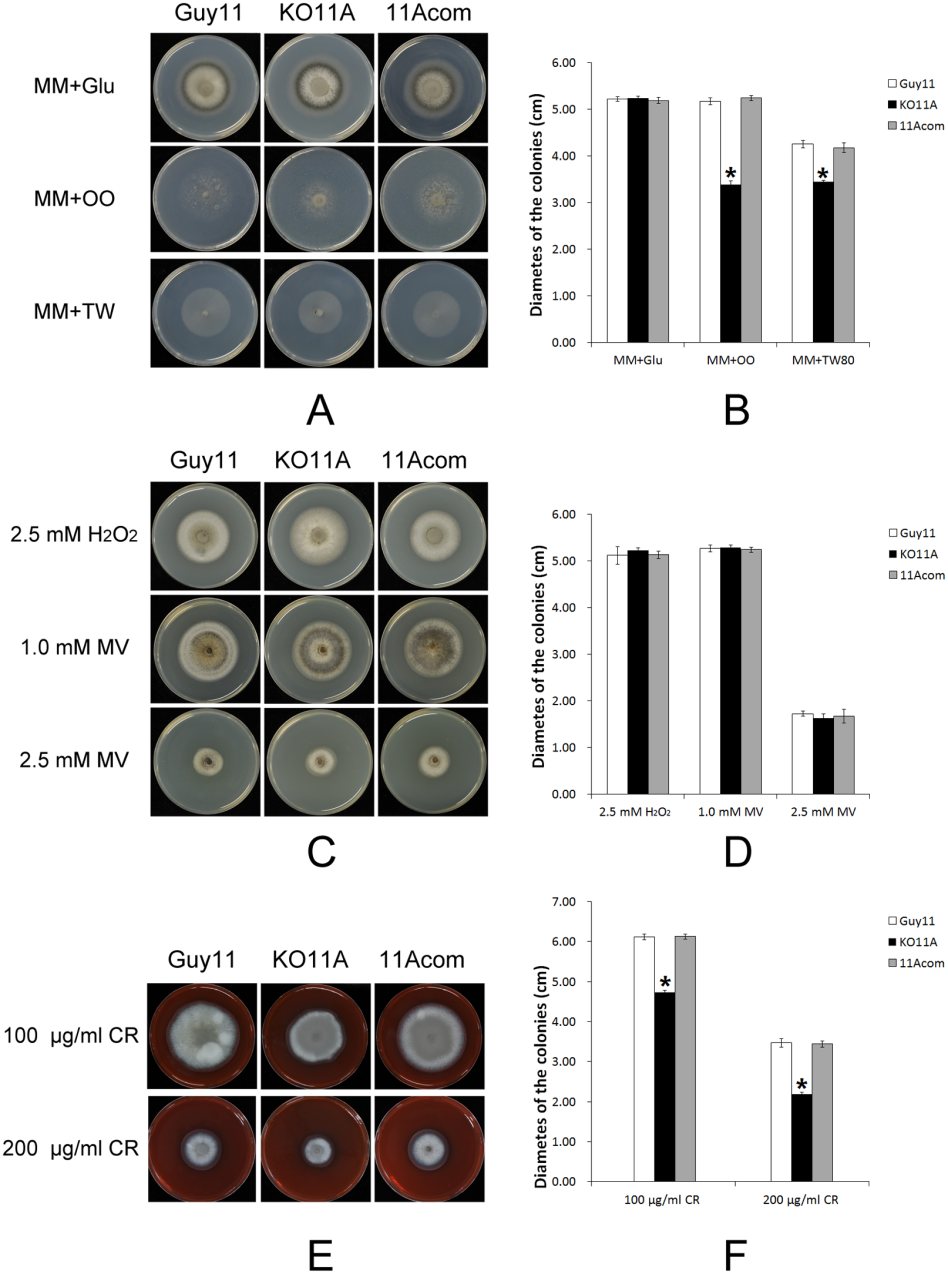
Lipid utilization and cell wall integrity test of the wild type and *MoPEX11* mutants. The strains were cultured on minimal medium with 1% Glucose, olive oil or Tween 80 as sole carbon source at 28°C for 8 d (A) and the colonial diameters were measured (B). The strains were cultured on CM supplemented with 200 μg/ml Congo red (CR) for 4 d (C), the colonial diameters were measured and the relative inhibition rates were compared (D). Standard deviations are indicated by the error bars. Asterisks indicate significant differences at p = 0.05.

As a result of the impacts to peroxisomal structures, the peroxisomal metabolisms were damaged by the deletion of *MoPEX11A*. On agar media with oleate as sole carbon source, the development of KO11A was significantly weakened, with smaller colonies and scarce aerial hyphae, in contrast with that of the wild type (Fig 6A and 6B). The experiments performed in liquid media with Tween 80 generated the identical results (S7 Fig), indicating the lipid degradation was disordered in the mutant. The acetyl-CoA generated from peroxisomal lipid oxidation is an important resource for glycerol accumulation in appressoria, thus the deficient lipid metabolism is likely a cause of the decrease in appressorial turgor in KO11A. The resistance to ROSs of the mutants was also compared by testing the growth on H_2_O_2_ and methyl viologen-containing media, but no significant deference was found (Fig 6C and 6D).

The acetyl-CoA is also an ingredient for cell wall synthesis [53], a key factor for appressorial morphogenesis and host infection of the fungus. On CM supplemented with Congo red (CR), the colonial diameter of KO11A was lower significantly than those of the wild type (Fig 6E and 6F), indicating the requirement of *MoPEX11A* in integrity of cell wall. These data suggest that *MoPEX11A* participate in the peroxisomal metabolisms of the fungus.

### Double and triple deletion of *MoPEX11A*, *B* and *C*

**Fig 7:**
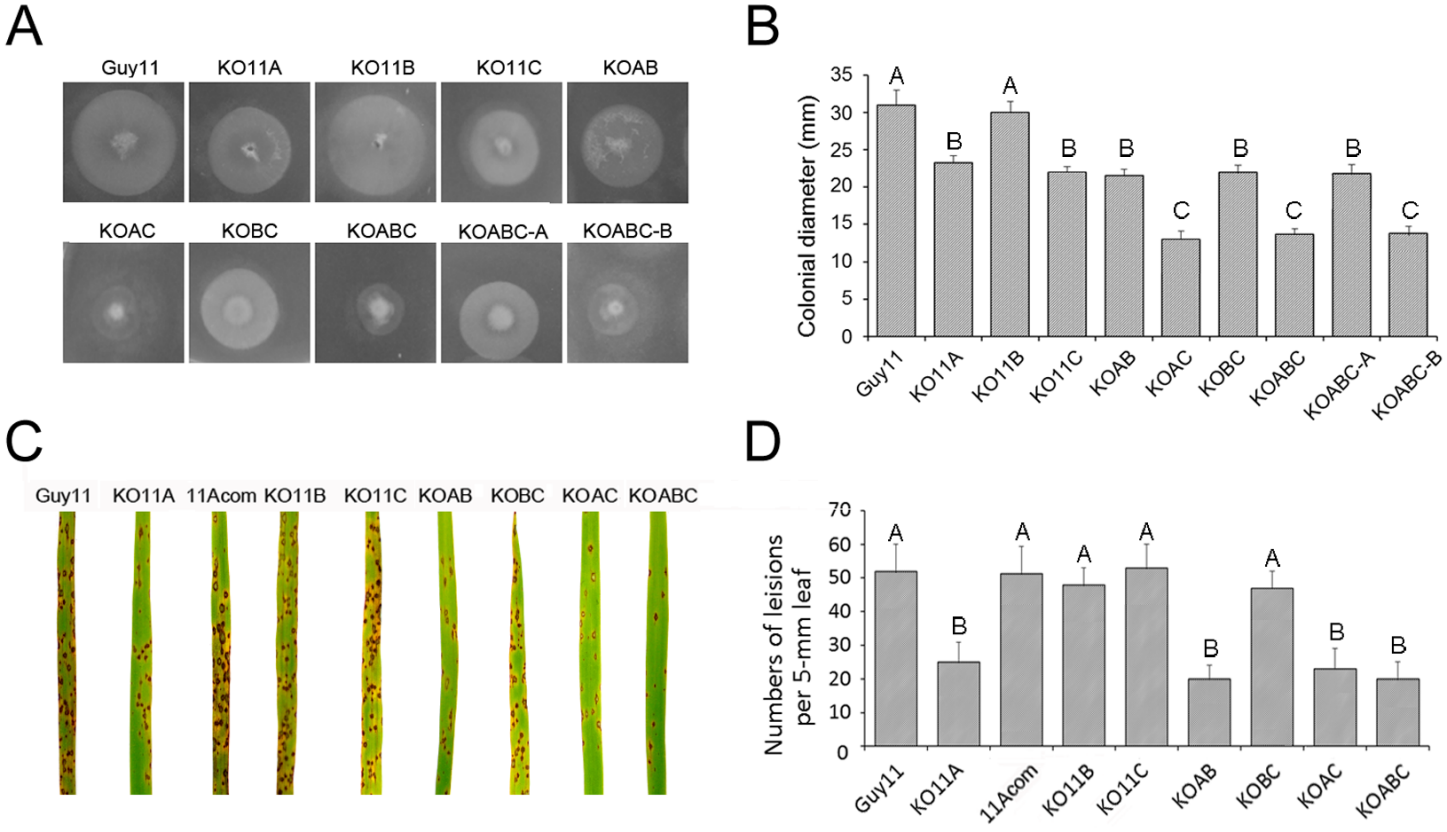
Lipid utilization and pathogenicity test of the single, double and triple mutants of *MoPEX11* genes. The strains were cultured on minimal medium with 1% Tween 80 as sole carbon source at 28°C for 5 d (A) and the colonial diameters were statistically compared (B). (C) Conidial suspension (1×10^5^ conidia/ml) of the strains were sprayed on 2-week-old rice cultivar CO39 and recorded at 7 dpi. (D) The numbers of lesions on 5-mm length leaf were counted and statistically analyzed. Different letters indicate significant differences between groups (p < 0.05).

To investigate whether the genes have synergistic effects, the double and triple deletions were performed. All the double and triple mutants grow well and form normal colonies on CM. On the media with oleate as sole carbon source, both KO11A and KO11C grew slower than the wild type, and KOAC grew slower than KO11A and KO11C. Namely, the double deletion of *MoPEX11A* and *MoPEX11C* aggravated the disorder in lipid metabolism, compared the single deletion of the each. The deletion of *MoPEX11B* cause neither the disorder in lipid utilization, and nor enhancement of the defects in KO11A or KO11C, since KO11B grew equally to the wild type, KOAB equally to KO11A, KOBC equally to KO11C, and KOABC equally to KOAC (Fig 7A and 7B). These results indicated *MoPEX11A* and *MoPEX11C* play roles synergistically in lipid degradation. However, the inoculation on rice seedlings indicated that although the mutants possessing *MoPEX11A* mutation, namely, KO11A, KO11AB, KO11AC and KO11ABC, generated remarkably reduced lesions compared with the wild type, the virulence of the four mutants are undifferentiated (Fig 7C and 7D). In addition, KO11B and KO11BC remained the equivalent virulence to the wild type. Additionally, the peroxisomes in all mutants are detectable by GFP-PTS1 visualization, indicating that the deletion of all the genes does not ruin the basal peroxisomal formation. The mutants harboring the deletion of *MoPEX11A* (KO11A, KO11AB, KO11AC and KOABC) form fewer and larger-sized peroxisomes than the wild type, but no significant difference was found between the mutants. Meanwhile, KO11BC had the equivalent peroxisomal profiles as KO11B, KO11C and the wild type. The results, together with those in the single mutants, confirmed that *MoPEX11A* is the major factor in mediating the peroxisomal number and sizes, *MoPEX11A* and *MoPEX11C* play roles in peroxisomal metabolism, while only *MoPEX11A* is indispensable for the pathogenicity.

### The impacts of deletion of *MoPEX11* genes to other related genes

**Fig 8:**
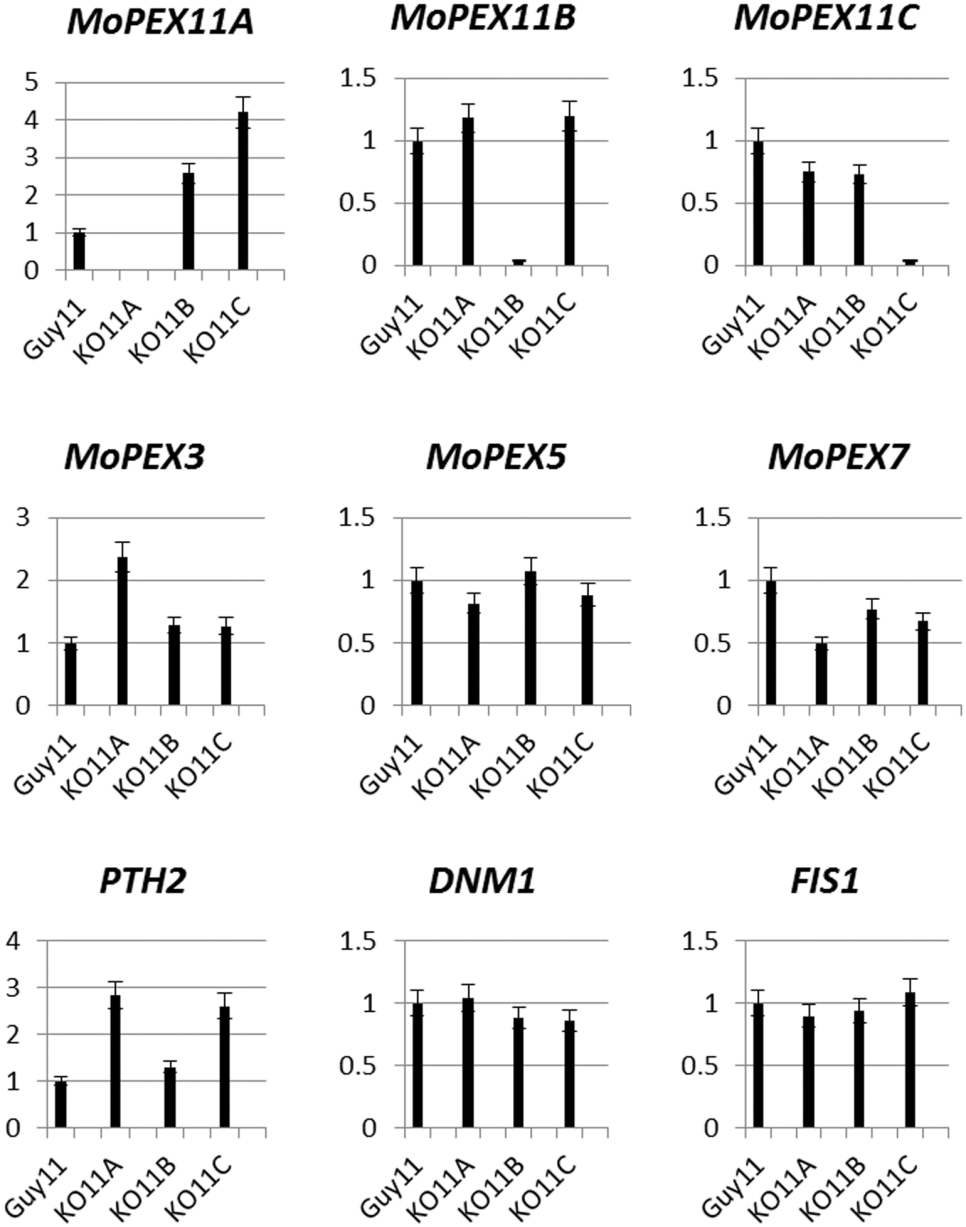
Impacts of the deletion of *MoPEX11* genes to the transcription of related genes. The relative abundance of the transcripts were detected by quantitative PCR with the total RNA from cultures on CM as template and β-tubulin gene (MGG00604) gene as internal reference. *MoPEX3*, MGG_06424; *DNM1*, MGG_06361; *FIS1*, MGG_06075.

To further explore the possible interactions of the genes, we checked the transcription of the genes in the mutants. The transcription of *MoPEX11A* was improved by the deletion of *MoPEX11B* or *MoPEX11C* (Fig 8). This data maybe implies a possibility that the defects caused by the deletion of *MoPEX11B* or *MoPEX11C* were partially offset by the improvement of *MoPEX11A*. The impacts of the *MoPEX11* genes to other peroxisome-related genes were also evaluated by transcriptional detection. *PTH2*, a gene encoding a carnitine aceryltransterase involved in fatty acid β-oxidation [32], was improved in KO11A and KO11C, consistent with the contributions of *MoPEX11A* and *MoPEX11C* in lipids metabolism. *MoPEX5* and *MoPEX7*, the 2 key genes involved in import of peroxisomal matrix proteins [34], only varied slightly in KO11A, indicating the peroxisomal import machinery was little impacted by *MoPEX11A*. However, *PEX3*, a gene required in peroxisomal de novo formation [54], was increasing transcribed in KO11A, reflecting that the fungus may improve the peroxisomal de novo formation when peroxisomal division is obstructed, to maintain the peroxisomal amount. The transcription of two fission factors, *DNM1* and *FIS1*, were not significantly affected by *MoPEX11A* deletion.

## Discussion

A specialty of Pex11 proteins is their multiplicity in one organism. The cytological and biochemical investigations demonstrated that five isoforms were present in *Arabidopsis thaliana* and three in mammals [55–58]. Three Pex11s were hypothesized in *M*. *oryzae*, basing on the sequences similarities, however, only Mopex11A of them exhibits high similarity to the Pex11s from plants or mammals. The functions of the Mopex11 proteins are thus unable to be speculated accurately via the sequence homology. The characterization of Pex11 isoforms in *P*. *chrysogenum* and Aopex11-1 in *A*. *oryzae* give valuable clues [39,40]. But because the numbers of Pex11 isoforms vary greatly in fungal species, it was still obscure which of and how the potential Mopex11 proteins are truly related to peroxisomal proliferation. In present work, we investigated the roles of the three Mopex11 proteins in peroxisomal proliferation and metabolism, giving the direct evidences for this subject.

Basing on the previous investigations, we hypothesize a typical Pex11, as a regulator of peroxisomal proliferation, has the capacity to alter peroxisomal number and size and to impact the peroxisomal metabolism [17]. The deletion of *MoPEX11A* significantly reduces the number of peroxisomes while increases their sizes. Meanwhile, lack of *MoPEX11A* greatly inhibits lipid utilization, and correspondingly, the transcription of *MoPEX11A* is up-regulated by lipids in the wild type strain. The lack of *MoPEX11C* did not significantly affect the peroxisomal profiles, but impacted the lipid metabolism, suggesting that Mopex11C may also have the capability to function as a peroxisomal proliferation regulator. In addition to Pex11A and Pex11C, ascomycetes also contain Pex11B. Our data showed the deletion of *MoPEX11B* affected neither the peroxisomal profiles nor the lipid metabolism. However, the transcription of *MoPEX11A* was found up-regulated in KO11B and KO11C strains, implying that a possibility that the fungus could offset the influences from the invalidation of *MoPEX11B* and *MoPEX11C* by improving the production of *MoPEX11A*, and which covered the defects partially in KO11B and KO11C. Thus, of the three potential Mopex11 proteins, Mopex11A is a key component of peroxisomal fission machinery, and Mopex11C may function as an assist, while Mopex11B may be nonfunctional or play unknown roles.

Woronin bodies were demonstrated to form by budding from the peroxisomes [52,59]. The major protein of Woronin bodies, Hex1, is synthesized in cytoplasm and imported into peroxisomes via routine peroxisomal import machinery, self-assembly inside peroxisomes, then buds and separates out to form the Woronin bodies coinciding with membrane fission [60]. GFP-MoHEX1 could be detected on peroxisomes in KO11A, in contrast to the cytoplasmic distribution in Δ*mopex5*, Δ*mopex6* and Δ*mopex19* mutants [37], indicating the peroxisomal import of Mohex1 is intact in the KO11A mutant. However, the independent localized Mohex1 were much less presented in KO11A than in the wild type, indicating that Mohex1 failed to divide and thus retarded inside the peroxisomes when *MoPEX11A* was deleted. The both requirement of *MoPEX11A* in peroxisomal proliferation and Woronin body differentiation suggested that the Woronin body formation shares not only the peroxisomal import pathways but also the Pex11 mediated peroxisomal fission machinery. Our data meanwhile demonstrated that Pex11B and Pex11C were not essential for the budding of Woronin bodies.

As a main metabolism, lipid degradation was strongly impaired by the deletion of the *PEX* genes involved in peroxisomal import in *M*. *oryzae*, *Podospora anserina*, *A*. *nidulans*, *Colletotrichum lagenarium* and *N*. *crassa* [32,42,60–63]. KO11A and KO11C decreased the growth on lipids, but in quite slight levels. Meanwhile, the resistance to ROSs of the *MoPEX11* mutants was unaltered, unlike that of the other *pex* mutants previously documented in *M*. *oryzae*. The weakness of cell wall was also a peroxisomal deficiency related phenotype. Δ*mopex5*, Δ*mopex6* and Δ*mopex19* were all hypersensitive to cell wall-perturbing agents Congo red and calcofluor white [32,34,37], whereas KO11A exhibited hypersensitivity only to Congo red, not to calcofluor white. These findings indicate the peroxisomal metabolism affected by the peroxisomal fission was not as greatly as the peroxisomal import.

The rice blast fungus *M*. *oryzae* is a model in investigation on plant fungal pathogens. Previous researches have demonstrated that peroxisome is indispensable for the infection of the fungus [32,34,37,41]. In these researches, the contributions of peroxisome to pathogenicity are generally related to lipid degradation, ROSs elimination and Woronin body formation. The Δ*mopex11A* strain caused reduced symptoms on rice and barley, whereas *MoPEX11B* and *MoPEX11C* likely play no roles in pathogenicity. The contributions of the three genes to pathogenicity correspond well to their performance in peroxisomal biogenesis and metabolism, confirming the importance of peroxisome in fungal pathogenicity. However, the reduction of the virulence in Δ*mopex11A* was apparently slighter than those in Δ*mopex5*, Δ*mopex6* or Δ*mopex19*, which lost the ability to cause disease completely [32,34,37]. Accordingly, Δ*mopex5*, Δ*mopex6* and Δ*mopex19* were deficient in melanization and turgor genesis of appressoria [32,34,41], while Δ*mopex11A* were only short of appressorial turgor. Thus the peroxisomal division likely impacts the fungal development and pathogenicity in relatively lower extent than peroxisomal import. This reminds of the possibility that the deficiency from peroxisomal fission was compensated, probably via the peroxisomal de novo formation. This speculation was supported by the fact that *PEX3*, a trigger protein of peroxisomal de novo formation [64], was increasingly transcribed in KO11A. In contrast, the transcription of *MoPEX5*, *MoPEX6* and *MoPEX7* which regulate the peroxisomal matrix import did not change significantly in KO11A, reflecting that the matrix import likely varies to adapt to the total peroxisomes, other than solely responds to peroxisomal fission. Another possibility of the fungus to offset the influences from disorders in peroxisomal fission was to overproduce the peroxisomal located enzymes, since the transcript level of *PTH2* which encodes carnitine aceryltransterase, a peroxisomal located enzyme playing key roles in fatty acids β-oxidation [32], was greatly improved in KO11A. The peroxisomes were found rapidly increasing at early stage of germination and appressorial differentiation of *M*. *oryzae* [46], and *MoPEX5* and *MoPEX7* exhibited accordant transcription dynamic which reached peak at 2 hpi [34]. *MoPEX11A* was also increasingly transcribed during this process, but its peak was present at 8 to 10 hpi. This indicates that the peroxisomal increase relies mainly on the de novo formation at early stage of appressorial differentiation, while the peroxisomal fission acts mainly at latter stages.

In summary, Mopex11A play major roles of the three potential Pex11 proteins in peroxisomal division, Mopex11C was likely as an assist, while Mopex11B play roles yet unknown. Peroxisomal proliferation was indispensable for the full virulence of *M*. *oryzae*, although less contributory than the peroxisomal matrix import.

## Supporting Information

S1 Fig Generation procedures of deletion mutants and complement transformants. The fungal strains were indicated in elliptic box, and the vector used were indicated in square box.
(TIF)

S2 Fig Sequence alignments of Pex11B (A) and Pex11C (B) proteins from fungal species. The identical amino acids are highlighted with black backgrounds, conserved residues with dark gray backgrounds, and similar amino acids with light gray backgrounds. Af, *A*. *fumigatus*; An, *A*. *nidulans*; Ao, *A*. *oryzae*; Gz, *Gibberella zeae*; Mo, *M*. *oryzae*; Nc, *N*. *crassa*; Pc, *P*. *chrysogenum*.
(TIF)

S3 Fig Deletion of *MoPEX11* genes and complementation of *Δmopex11A* mutant. (A) Diagram and Southern blotting indicative replacement of *MoPEX11A*. DNA samples were digested with *Bst*X I and hybridized with the probe indicated. A 3471-bp hybridization band was detected in the wild type, whereas 5563-bp bands in the mutants. (B) Diagram and Southern blotting indicative *MoPEX11B* replacement. DNA samples were digested with *Sac* I. A 5408-bp hybridization band was detected in the wild type while 6921-bp bands in the mutants. (C) Diagram and Southern blotting indicative *MoPEX11C* replacement. DNA samples were digested with *Sal* I. A 3987-bp band was detected in the wild type whereas 6413-bp bands in the mutants. (D) Transcription analysis by quantitative PCR to confirm the gene deletion in *Δmopex11A* mutant (KO11A) and regain in the complementary strain (11A-com). *MoPEX11A* transcripts were detected in similar abundance in 11A-com and the wild type (Guy11), but undetectable in KO11A.
(TIF)

S4 Fig The vegetative growth of *MoPEX11* mutants and the wild type. (A) The strains were cultured on CM at 28°C for 5 days. Statistically comparison of the radial growth (B) and conidiation per petri dish (C). Means and standard errors were calculated from three independent replicates.
(TIF)

S5 Fig Pathogenicity related morphogenesis of the wild type and the *MoPEX11* mutants. The conidia harvested from 10-day-old complete media were incubated on inducible plastic membrane and the germination rates (A) and appressorial formation rates (B) of the strains were calculated at time points. (C) The 24h appressoria formed by the wild type, KO11A and the complementary strain. Bar = 5 μm. (D) The turgor generation of the 24 h appressoria was evaluated by counting the collapse rate in glycerol in gradient concentrations. Standard deviations are indicated by the error bars. Asterisks indicate significant differences at p = 0.05.
(TIF)

S6 Fig CLSM analysis of peroxisomes profiles visualized with DsRed-PTS1 (A) and GFP- PTS2 (B) in the wild type and the *MoPEX11* mutants. Bars = 5 μm.
(TIF)

S7 Fig Biomass quantification of the wild type and *MoPEX11A* mutant cultured in liquid lipid media. The conidia of the strains were suspended in minimal medium with 1% Glucose or Tween 80 as sole carbon source at 1 × 10^6^ conidia/ml shaking at 150 rpm at 28°C for 4 d. The cultures were filtrated to remove the supernatant, dried at 37°C in a drying oven, and then weighed and compared. Standard deviations are indicated by the error bars. Asterisk indicate the significant difference at p = 0.05 to the wild type cultured at same conditions.
(TIF)
