# Supplementary figures and images for: One of Three Pex11 Family Members Is Required for Peroxisomal Proliferation and Full Virulence of the Rice Blast Fungus Magnaporthe oryzae
Source: PLoS One. 2015 Jul 28;10(7):e0134249. doi: 10.1371/journal.pone.0134249 (PMC4517885; doi:10.1371/journal.pone.0134249)

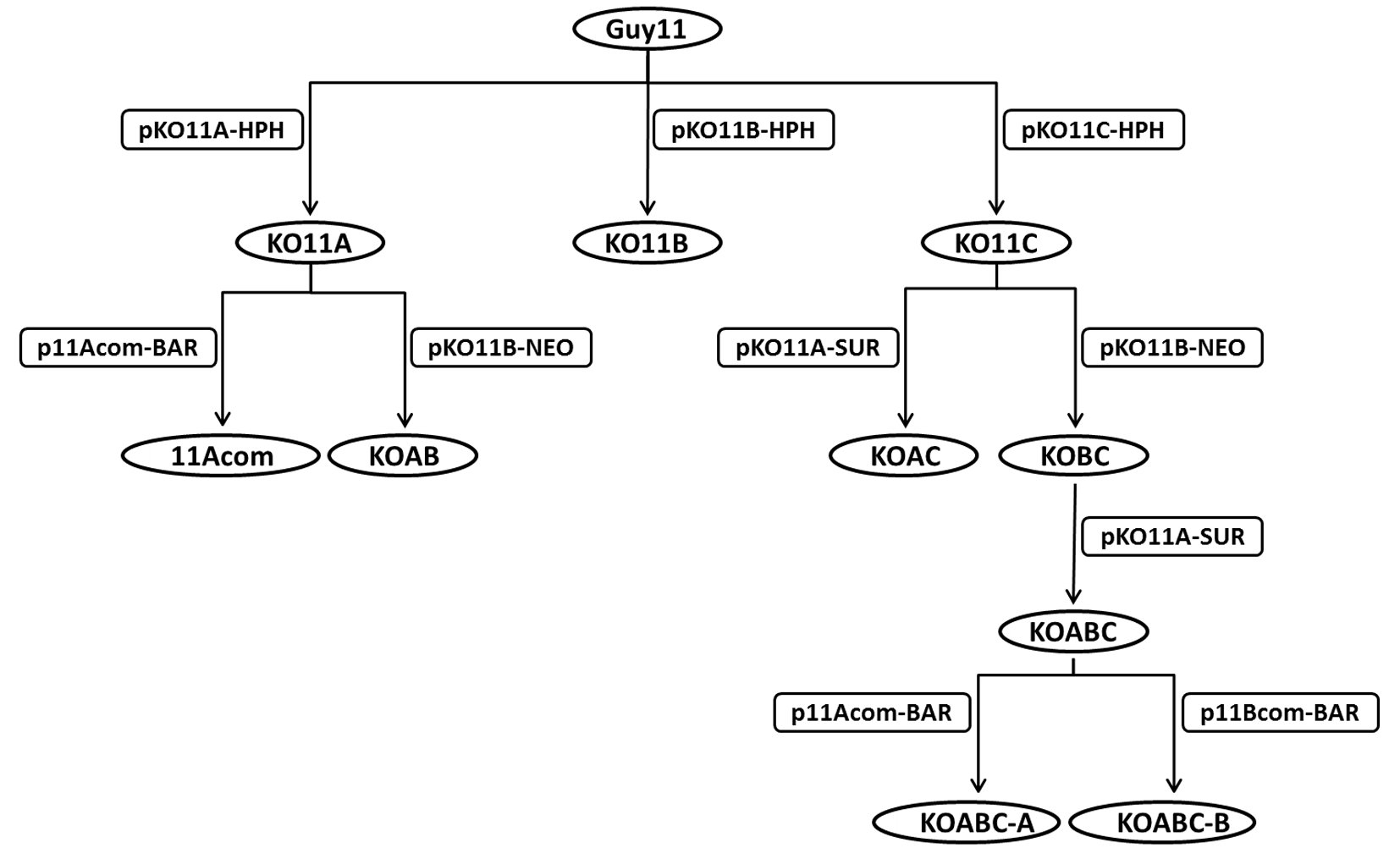

Supplement: S1 Fig — The fungal strains were indicated in elliptic box, and the vector used were indicated in square box. (TIF) [file pone.0134249.s001.tif]

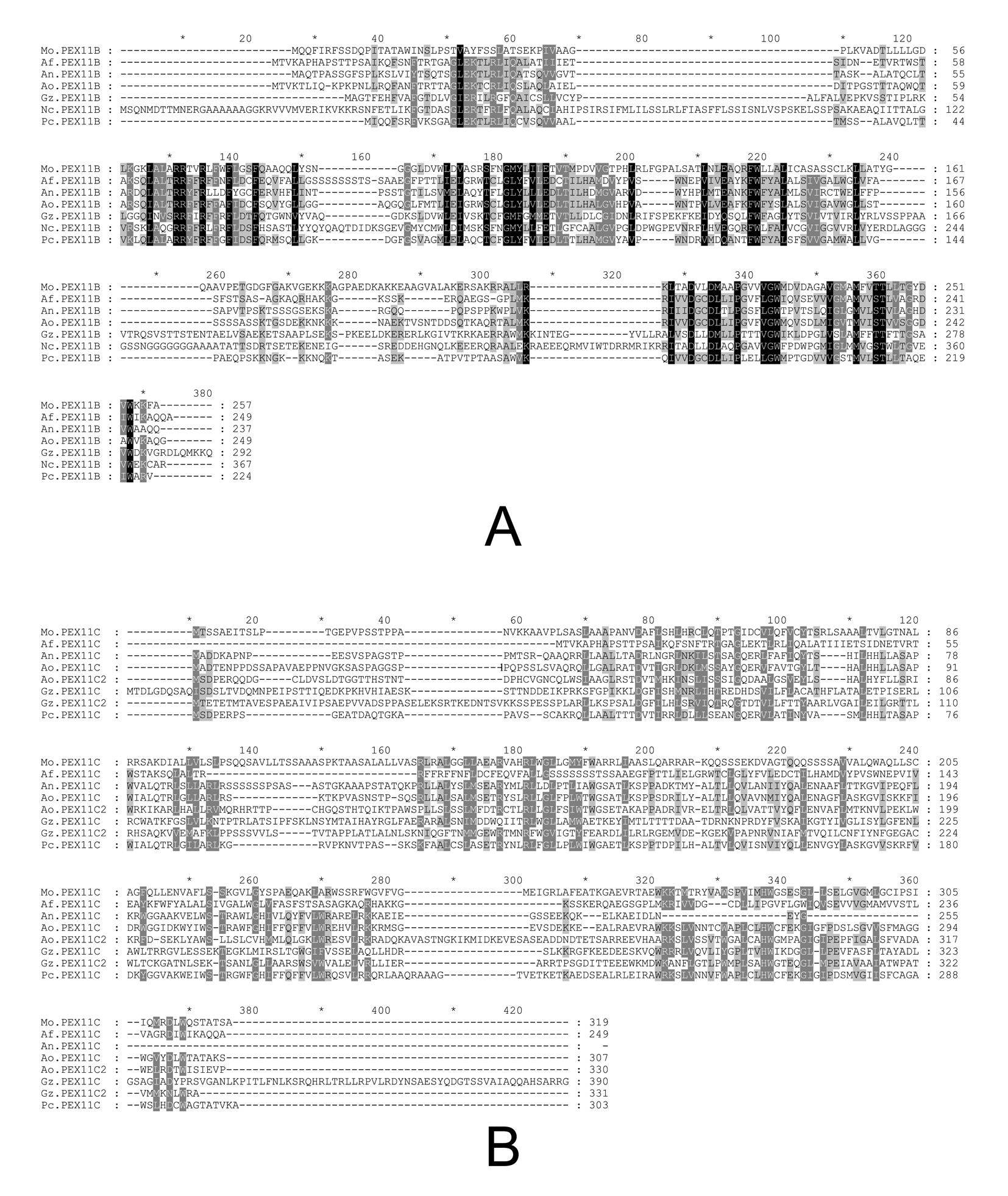

Supplement: S2 Fig — The identical amino acids are highlighted with black backgrounds, conserved residues with dark gray backgrounds, and similar amino acids with light gray backgrounds. Af, A. fumigatus; An, A. nidulans; Ao, A. oryzae; Gz, Gibberella zeae; Mo, M. oryzae; Nc, N. crassa; Pc, P. chrysogenum. (TIF) [file pone.0134249.s002.tif]

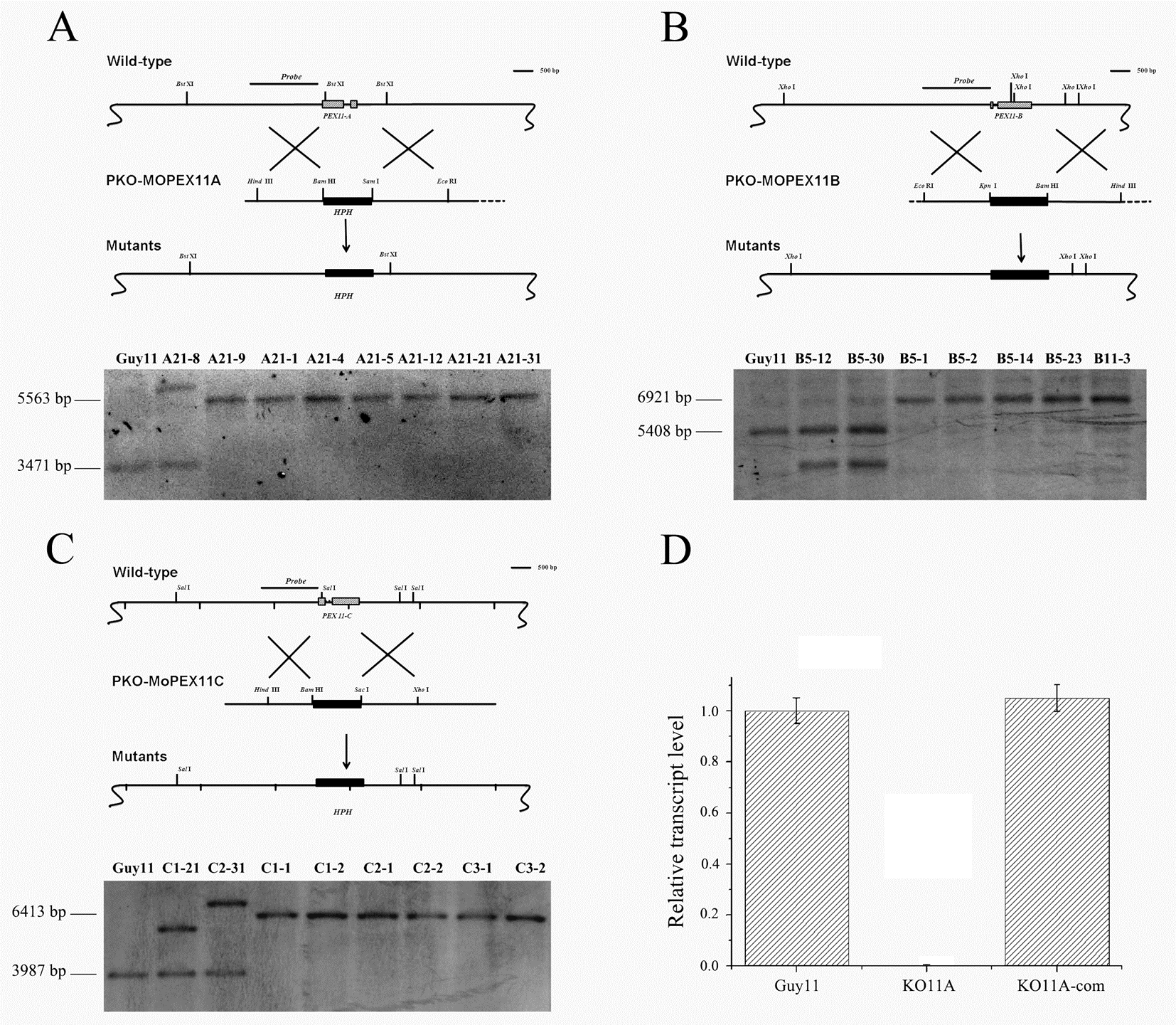

Supplement: S3 Fig — (A) Diagram and Southern blotting indicative replacement of MoPEX11A. DNA samples were digested with BstX I and hybridized with the probe indicated. A 3471-bp hybridization band was detected in the wild type, whereas 5563-bp bands in the mutants. (B) Diagram and Southern blotting indicative MoPEX11B replacement. DNA samples were digested with Sac I. A 5408-bp hybridization band was detected in the wild type while 6921-bp bands in the mutants. (C) Diagram and Southern blotting indicative MoPEX11C replacement. DNA samples were digested with Sal I. A 3987-bp band was detected in the wild type whereas 6413-bp bands in the mutants. (D) Transcription analysis by quantitative PCR to confirm the gene deletion in Δmopex11A mutant (KO11A) and regain in the complementary strain (11A-com). MoPEX11A transcripts were detected in similar abundance in 11A-com and the wild type (Guy11), but undetectable in KO11A. (TIF) [file pone.0134249.s003.tif]

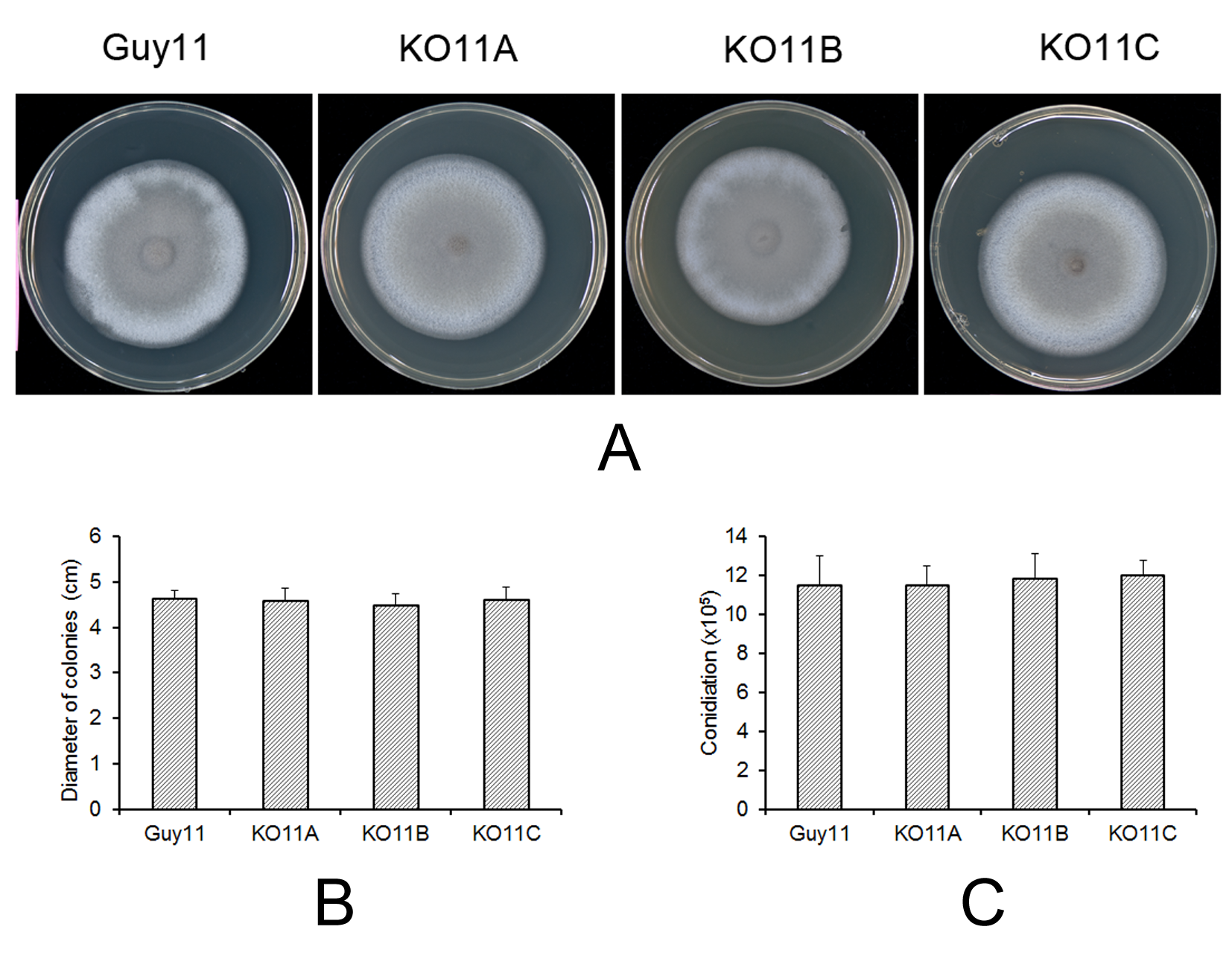

Supplement: S4 Fig — (A) The strains were cultured on CM at 28°C for 5 days. Statistically comparison of the radial growth (B) and conidiation per petri dish (C). Means and standard errors were calculated from three independent replicates. (TIF) [file pone.0134249.s004.tif]

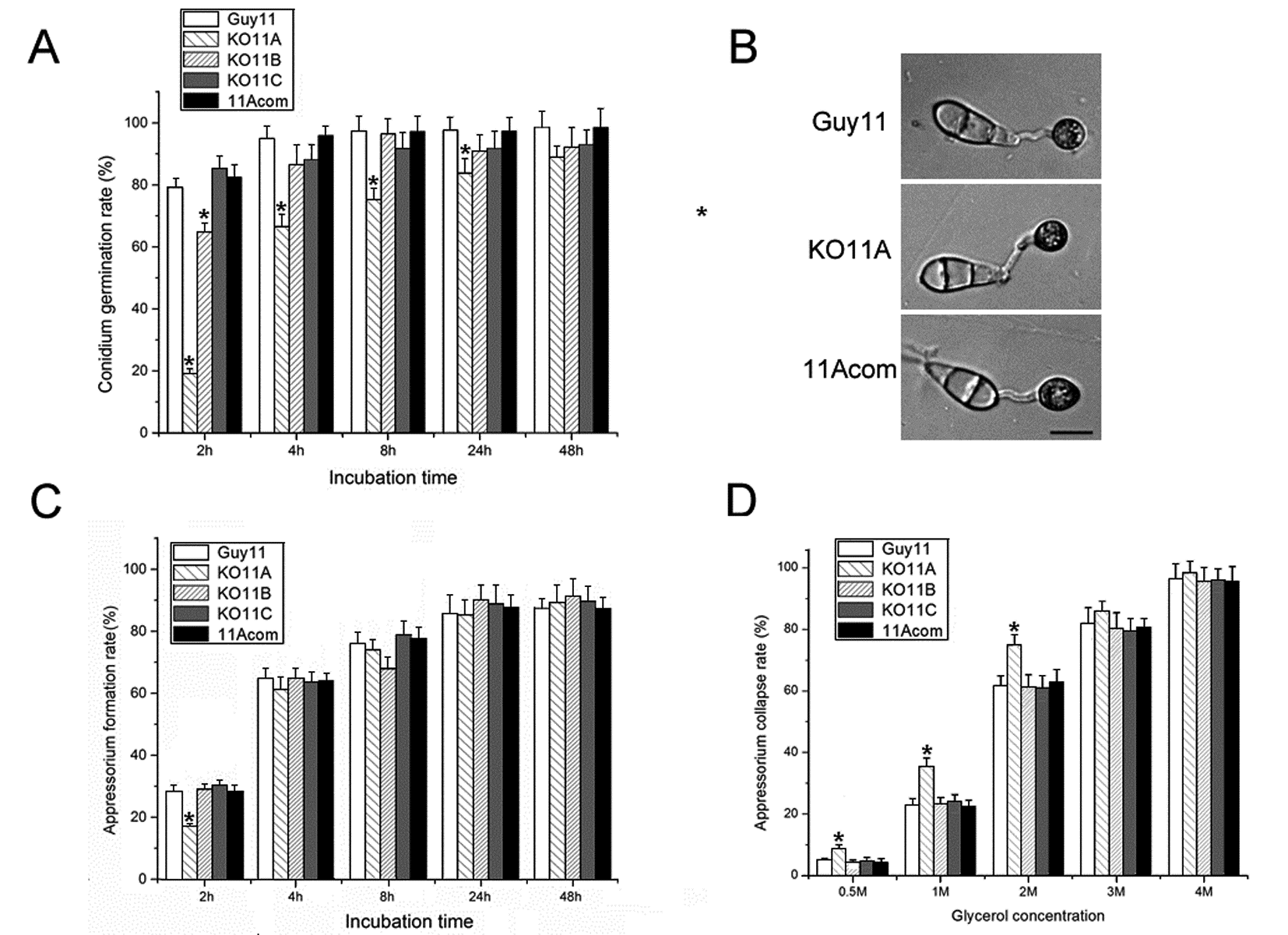

Supplement: S5 Fig — The conidia harvested from 10-day-old complete media were incubated on inducible plastic membrane and the germination rates (A) and appressorial formation rates (B) of the strains were calculated at time points. (C) The 24h appressoria formed by the wild type, KO11A and the complementary strain. Bar = 5 μm. (D) The turgor generation of the 24 h appressoria was evaluated by counting the collapse rate in glycerol in gradient concentrations. Standard deviations are indicated by the error bars. Asterisks indicate significant differences at p = 0.05. (TIF) [file pone.0134249.s005.tif]

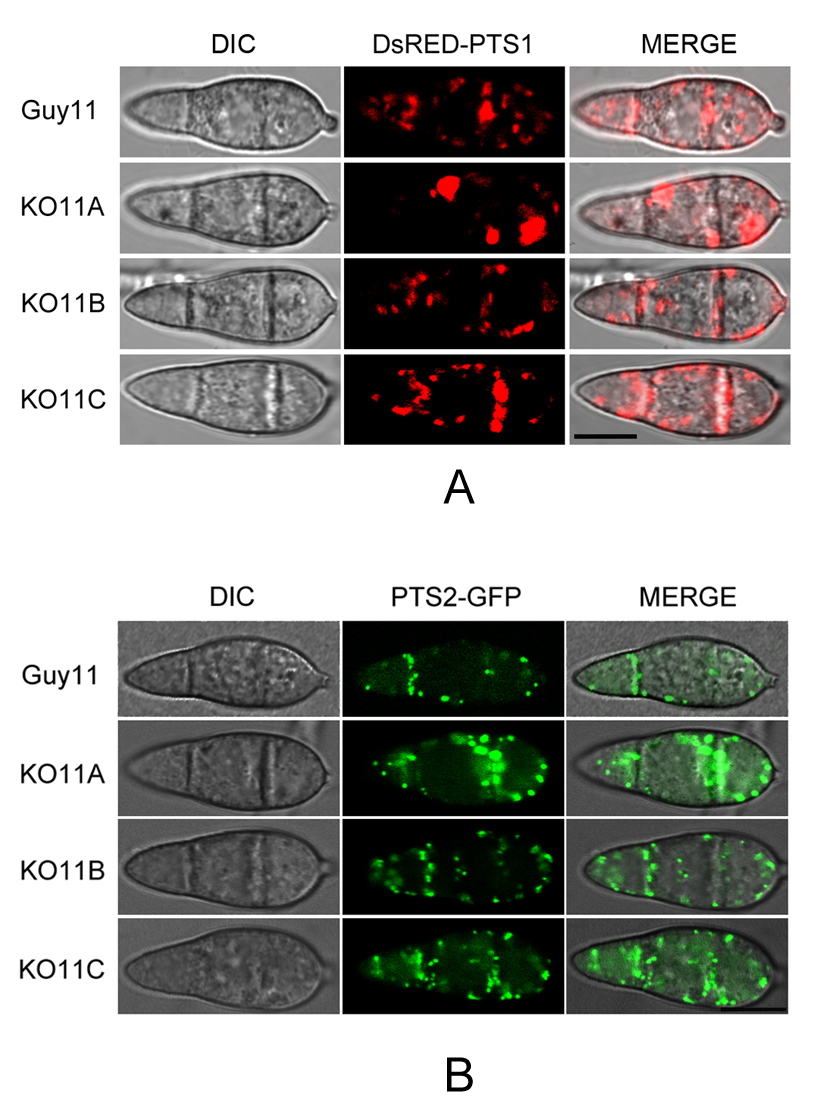

Supplement: S6 Fig — Bars = 5 μm. (TIF) [file pone.0134249.s006.tif]

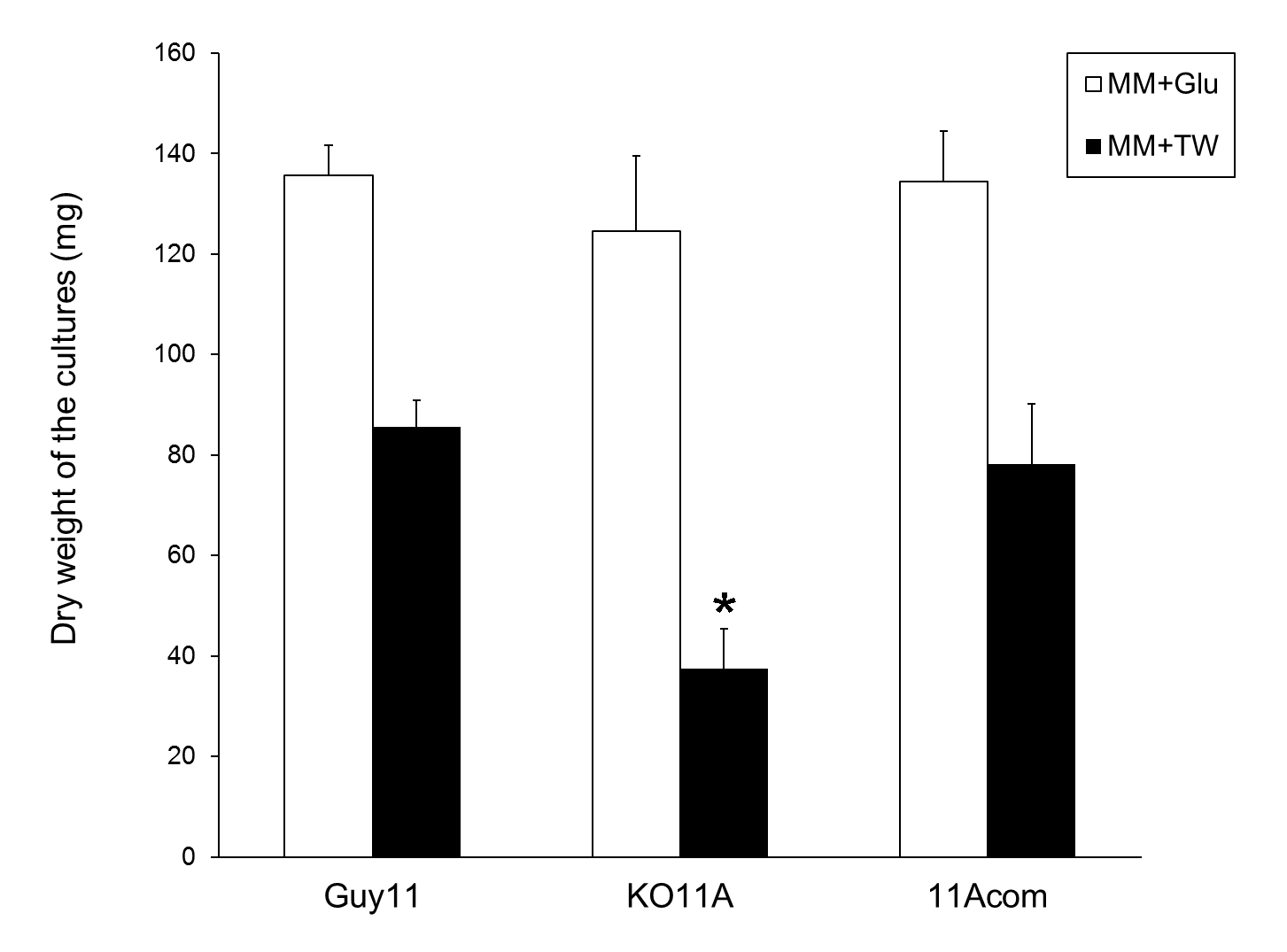

Supplement: S7 Fig — The conidia of the strains were suspended in minimal medium with 1% Glucose or Tween 80 as sole carbon source at 1 × 10^6^ conidia/ml shaking at 150 rpm at 28°C for 4 d. The cultures were filtrated to remove the supernatant, dried at 37°C in a drying oven, and then weighed and compared. Standard deviations are indicated by the error bars. Asterisk indicate the significant difference at p = 0.05 to the wild type cultured at same conditions. (TIF) [file pone.0134249.s007.tif]
